# Supplementary material for: A whole-animal phenotypic drug screen identifies suppressors of atherogenic lipoproteins
Source: eLife. 2026 Aug 27;14:RP105314. doi: 10.7554/eLife.105314 (PMC13521566; doi:10.7554/eLife.105314)
Supplement: Supplementary file 1 [file elife-105314-mdarchecklist1.docx]

**Materials Design Analysis Reporting (MDAR) Checklist for Authors**

*Kelpsch DJ, Zhang L, Thierer JH, Rivera Cruz AG, Koren K, Kumar U, Lin Y, Hensley MR, Sohn M, Liu JO, Lectka T, Mumm JS, Farber SA. "A whole-animal phenotypic drug screen identifies suppressors of atherogenic lipoproteins."*

**Materials:**

| **Newly created materials** | **Indicate where provided: section/figure legend** | **N/A** |
| --- | --- | --- |
| The manuscript includes a dedicated "materials availability statement" providing transparent disclosure about availability of newly created materials including details on how materials can be accessed and describing any restrictions on access. | Materials and Methods, "Zebrafish husbandry and maintenance", and Key Resources Table. |  |

| **Antibodies** | **Indicate where provided: section/figure legend** | **N/A** |
| --- | --- | --- |
| For commercial reagents, provide supplier name, catalogue number and RRID, if available. |  | N/A |

| **DNA and RNA sequences** | **Indicate where provided: section/figure legend** | **N/A** |
| --- | --- | --- |
| Short novel DNA or RNA including primers, probes: Sequences should be included or deposited in a public repository. |  | N/A |

| **Cell materials** | **Indicate where provided: section/figure legend** | **N/A** |
| --- | --- | --- |
| Cell lines: Provide species information, strain. Provide accession number in repository OR supplier name, catalog number, clone number, OR RRID. |  | N/A |
| Primary cultures: Provide species, strain, sex of origin, genetic modification status. |  | N/A |

| **Experimental animals** | **Indicate where provided: section/figure legend** | **N/A** |
| --- | --- | --- |
| Laboratory animals or Model organisms: Provide species, strain, sex, age, genetic modification status. Provide accession number in repository OR supplier name, catalog number, clone number, OR RRID. | Materials and Methods, "Zebrafish husbandry and maintenance" and "Characterizing lipoproteins in HNF4⍺ mutants"; Key Resources Table.  Species: Danio rerio. Lines: Fus(ApoBb.1-NanoLuciferase) ("LipoGlo", Thierer et al., 2019); hnf4a rdu14 (Davison et al., 2017, obtained from the J.F. Rawls laboratory). Genetic modification status: endogenous NanoLuciferase knock-in, transgenic reporter, and germline loss-of-function allele, respectively. Age: 1–5 days post fertilization for all experiments. Sex: zebrafish sex is not determined until the juvenile stage, so sex is not a variable in these embryonic and larval experiments (stated in Materials and Methods). |  |
| Animal observed in or captured from the field: Provide species, sex, and age where possible. |  | N/A |

| **Plants and microbes** | **Indicate where provided: section/figure legend** | **N/A** |
| --- | --- | --- |
| Plants: provide species and strain, ecotype and cultivar where relevant, unique accession number if available, and source (including location for collected wild specimens). |  | N/A |
| Microbes: provide species and strain, unique accession number if available, and source. |  | N/A |

| **Human research participants** | **Indicate where provided: section/figure legend, or state if these demographics were not collected** | **N/A** |
| --- | --- | --- |
| If collected and within the bounds of privacy constraints report on age, sex, gender and ethnicity for all study participants. |  | N/A |

**Design:**

| **Study protocol** | **Indicate where provided: section/figure legend** | **N/A** |
| --- | --- | --- |
| If the study protocol has been pre-registered, provide DOI. For clinical trials, provide the trial registration number OR cite DOI. |  | N/A |

| **Laboratory protocol** | **Indicate where provided: section/figure legend** | **N/A** |
| --- | --- | --- |
| Provide DOI OR other citation details if detailed step-by-step protocols are available. | The ARQiv-HTS automated screening platform is described in full in White et al., 2016, Nature Protocols (DOI:10.1038/nprot.2016.142). The LipoGlo assay and LipoGlo electrophoresis methods are described in Thierer et al., 2019 (DOI:10.1038/s41467-019-11259-w). Both are cited in Materials and Methods. |  |

| **Experimental study design (statistics details)**  **For in vivo studies: State whether and how the following have been done** | **Indicate where provided: section/figure legend. If it could have been done, but was not, write "not done"** | **N/A** |
| --- | --- | --- |
| Sample size determination | Yes — see Results, "A whole-animal drug screen to identify B-lp-reducing compounds": eight independent replicates per treatment condition, selected on the basis of variation observed in the B-lp assay in prior experiments. |  |
| Randomisation | Formal randomization was not done. |  |
| Blinding | For the HNF4⍺ genotype experiments, samples were de-identified by genotyping after data collection (Materials and Methods, "Characterizing lipoproteins in HNF4⍺ mutants"). Drug blinding was performed for the primary screen but not drug validation assays. |  |
| Inclusion/exclusion criteria | Yes — pre-established. Hits were defined a priori as compound doses with SSMD < −1.0 and fold change < −1.5 (log2 scale), following Zhang, 2007 and Zhang, 2011 (Results and Materials and Methods, "Quantification and statistical analysis").  Outliers were identified using Tukey fences (1.5 × interquartile range), computed within each treatment group of each independent experiment, and removed prior to statistical testing. Applied to validation assays, homogenate assays, and native-PAGE quantification. |  |

| **Sample definition and in-laboratory replication** | **Indicate where provided: section/figure legend** | **N/A** |
| --- | --- | --- |
| State number of times the experiment was replicated in the laboratory. | Primary screen: 1381 96-well plates, each containing its own vehicle and 5 µM lomitapide controls. Validation assays (fixation and homogenization endpoints) and NanoLuciferase activity assays: three independent experiments each (Materials and Methods). Secondary characterization in Figure 3: two (3A), three (3B, 3D, 3F, 3H) or four (3C) independent experiments, as stated in the legend. Native-PAGE: three independent experiments (Figures 3, 4), two for Figure 5D. RNAseq: three independent clutches per treatment and duration. HNF4⍺ developmental series: at least three independent experiments; Figure 6B: two independent experiments. |  |
| Define whether data describe technical or biological replicates. | Both are defined and distinguished. In the primary screen each well contained a single larva, so the eight replicates per condition are biological replicates. In validation assays, compounds were tested with one to three biological replicates, each comprising eight technical replicates (Materials and Methods, "LipoGlo assays with fixation endpoint"). In homogenate assays each data point is an individual animal. Sample sizes (n) are given in every figure legend and supplemental figure. |  |

| **Ethics** | **Indicate where provided: section/submission form** | **N/A** |
| --- | --- | --- |
| Studies involving human participants: State details of authority granting ethics approval (IRB or equivalent committee(s), provide reference number for approval. |  | N/A |
| Studies involving experimental animals: State details of authority granting ethics approval (IRB or equivalent committee(s), provide reference number for approval. | Materials and Methods, "Zebrafish husbandry and maintenance": all zebrafish protocols were approved by the Carnegie Institution Department of Embryology Animal Care and Use Committee, Protocol #139. Also entered in the submission form. |  |
| Studies involving specimen and field samples: State if relevant permits obtained, provide details of authority approving study; if none were required, explain why. |  | N/A |

| **Dual Use Research of Concern (DURC)** | **Indicate where provided: section/submission form** | **N/A** |
| --- | --- | --- |
| If study is subject to dual use research of concern regulations, state the authority granting approval and reference number for the regulatory approval. |  | N/A |

**Analysis:**

| **Attrition** | **Indicate where provided: section/figure legend** | **N/A** |
| --- | --- | --- |
| Describe whether exclusion criteria were pre-established. Report if sample or data points were omitted from analysis. If yes, report if this was due to attrition or intentional exclusion and provide justification. | Exclusion criteria were pre-established (SSMD < −1.0 and fold change < −1.5, log2 scale). Intentional exclusions are reported in Results and Supplemental Table 3: of 49 unique hits, one compound could not be identified and 11 were not obtained or validated; gentian violet was lethal at all doses tested and yielded no quantifiable data; verteporfin was excluded from the final set of 19 leads because it directly inhibits NanoLuciferase. Compounds falling below the SSMD and fold-change cutoffs were not re-tested, and this limitation is stated in the Discussion.  Individual data points were excluded as outliers using Tukey fences (1.5 × interquartile range), computed within each treatment group of each independent experiment. This was an intentional, pre-established exclusion rather than attrition, and was applied to validation assays, homogenate assays, and native-PAGE quantification only. Fewer than 6% of data points were excluded in any individual experiment. The sample sizes (n) reported in every figure legend and supplemental figure are post-exclusion counts. |  |

| **Statistics** | **Indicate where provided: section/figure legend** | **N/A** |
| --- | --- | --- |
| Describe statistical tests used and justify choice of tests. | Materials and Methods, "Quantification and statistical analysis". Screen data were analyzed by fold change and Strictly Standardized Mean Difference (SSMD), chosen because SSMD penalizes fold change by its variability and is robust to variation and outliers in high-replicate screens (Zhang, 2007; Zhang, 2011); cutoffs follow those authors. Remaining experiments used one-way ANOVA followed by Dunnett’s test against the vehicle control with Bonferroni correction for multiple comparisons; Figure 6 used two-way ANOVA to test genotype, treatment, and their interaction. Exact p-values, F-statistics with degrees of freedom, and n are reported in each figure legend. |  |

| **Data availability** | **Indicate where provided: section/submission form** | **N/A** |
| --- | --- | --- |
| For newly created and reused datasets, the manuscript includes a data availability statement that provides details for access (or notes restrictions on access). | Raw primary screen data are provided as Supplemental Table 1 and analyzed summary data as Supplemental Table 2. All raw data and analysis code is provided as Source Code 1. |  |
| When newly created datasets are publicly available, provide accession number in repository OR DOI and licensing details where available. | Primary screen data are supplied in full as Supplemental Tables 1 and 2. |  |
| If reused data is publicly available provide accession number in repository OR DOI, OR URL, OR citation. | Differentially expressed gene lists from HNF4⍺, HNF4Ɣ, and HNF4⍺/HNF4Ɣ double knockout zebrafish intestines were reused for the comparison in Figure 7E and Supplemental Table 7, from Heppert et al., 2022, Genetics (DOI:10.1093/genetics/iyac133).  Prochlorperazine transcriptional signatures (MCF7, PC3) were accessed through Enrichr (Chen et al., 2013; Kuleshov et al., 2016; Xie et al., 2021). |  |

| **Code availability** | **Indicate where provided: section/figure legend** | **N/A** |
| --- | --- | --- |
| For any computer code/software/mathematical algorithms essential for replicating the main findings of the study, whether newly generated or re-used, the manuscript includes a data availability statement that provides details for access or notes restrictions. | Materials and Methods, "Quantification and statistical analysis": all data processing and analysis used custom R scripts, provided with the manuscript as Source Code 1. |  |
| Where newly generated code is publicly available, provide accession number in repository, OR DOI OR URL and licensing details where available. State any restrictions on code availability or accessibility. | Custom analysis code is supplied with the manuscript as Source Code 1; no restrictions apply. |  |
| If reused code is publicly available provide accession number in repository OR DOI OR URL, OR citation. | All are cited in Materials and Methods and listed in the Key Resources Table. |  |

**Reporting:**

The MDAR framework recommends adoption of discipline-specific guidelines, established and endorsed through community initiatives.

| **Adherence to community standards** | **Indicate where provided: section/figure legend** | **N/A** |
| --- | --- | --- |
| State if relevant guidelines (e.g., ICMJE, MIBBI, ARRIVE, STRANGE) have been followed, and whether a checklist (e.g., CONSORT, PRISMA, ARRIVE) is provided with the manuscript. |  | N/A |
