# Supplementary material for: A whole-animal phenotypic drug screen identifies suppressors of atherogenic lipoproteins
Source: eLife. 2026 Aug 27;14:RP105314. doi: 10.7554/eLife.105314 (PMC13521566; doi:10.7554/eLife.105314)
Supplement: Peer review history

# Editor report: eLife Assessment

**DOI:** 10.7554/eLife.105314.3.sa0

**Authors:** Richard M White

**Affiliations:** University of Oxford United Kingdom

**Keywords:** Convincing, Important

In this **important** study, the authors have performed a zebrafish drug screen to identify suppressors of atherogenic lipoproteins. They utilize a well-established LipoGlo assay to find molecules that modulate these lipoproteins, identifying 49 potential hits and they perform validation experiments, including studies linking enoxolone to its likely inhibitory effect on a specific transcription factor, HNF4alpha. Overall, the results are **convincing** and robust, and will open up new areas of exploration for those investigators interested in in vivo lipid biology.

# Referee report: Reviewer #1 (Public review)

**DOI:** 10.7554/eLife.105314.3.sa1

[Editors' note: The authors addressed reviewer comments well, further strengthening the conclusions of the study.]

Summary:

A whole-organism drug screen was performed to identify molecules that decrease Apolipoprotein B (ApoB) as a target for agents to reduce atherosclerosis. Kelpsch et al. used a zebrafish reporter line, LipoGlo, which is a fusion of the Nano-luciferase protein to the ApoB protein as a proxy for the presence of ApoB-containing lipoproteins (B-lps) in larval stages. The LipoGlo line was screened against a well-characterized drug library and identified 49 hits from their primary screen. Follow-up studies further refined this list to 19 molecules that reproducibly reduced B-lps significantly. The authors focused their studies on enoxolone, a licorice root extract, and showed that larvae treated with this agent can reduce the production of B-lps. As enoxolone has been reported to suppress Hepatocyte Nuclear factor 4a (HNF4a), the authors investigated whether loss-of-hnf4a or pharmacological inhibition of hnf4a in zebrafish also produced similar phenotypes as enoxolone treatment. Their studies showed that this was the case. Transcriptomic studies after enoxolone treatment resulted in altered expression of genes involved in cholesterol biosynthesis and in glucose/insulin signaling pathways. This study highlights the utility of a zebrafish whole-organism chemical screen for modifiers of B-lps production and/or its clearance. A significant finding is that enoxolone inhibits hnf4a in zebrafish to reduce B-lps production and supports targeting HNF4a as a therapeutic means to reduce the emergence of atherosclerosis.

Strengths:

The authors performed a whole-organism chemical screen with over 3000 agents. Such screens are challenging, and the authors used strict criteria for determining hits. The conclusions of this study are well supported by the presented data.

Comment on revised version:

The authors have addressed all my comments.

# Referee report: Reviewer #2 (Public review)

**DOI:** 10.7554/eLife.105314.3.sa2

Summary:

The authors aimed to develop a large-scale drug screen to identify B-lp modulators in a vertebrate whole-animal system. Using the zebrafish LipoGlo system that the authors had previously published and validated, the authors screened 2762 drug candidates to generate 49 hits and ultimately validated 19 drugs as genuine ApoB-lowering drugs. Using LipoGlo-Electrophoresis, the authors are able to obtain insights into the ApoB-lipoprotein size/subclass distribution. The authors further validate and study the mechanism of a strong hit, Enoxolone, known as also known as 18β-Glycyrrhetinic acid, which has previously been reported to modulate lipid metabolism. The authors also show that Enoxolone effects are mediated through HNF4⍺, which has been previously shown in the mouse system, but this is the first time it has been shown in the zebrafish.

Strengths:

The study was methodical and robust, using a published and well-validated zebrafish LipoGlo model. The authors validated the hits from the screen independently and considered the possibility that some drugs may have been detected as false positive results due to effects on the enzymatic activity of NanoLuciferase; only one hit, verteporfin, was shown to be a false positive. Using LipoGlo-Electrophoresis, the authors are able to obtain extra insights into the ApoB-lipoprotein size/subclass distribution. They showed that while enoxolone treatment reduces total B-lps, there are no overt changes in B-lp size distribution compared to vehicle-treated animals, other than a slight increase in the zero mobility (ZM) fraction, which contains very large particles and/or tissue aggregates. In contrast, the positive control, lomitapide, does show a change in B-lp size distribution compared to vehicle-treated animals - an increase in frequency of LDLs (low-density lipoprotein), but a decrease in VLDLs (very low-density lipoprotein). This study also assesses the LipoGlo-Electrophoresis profile of HNF4⍺ inhibitors. Work in the zebrafish larvae means that the effect on overall development and an entire vertebrate organism can also be assessed. Finally, the authors applied a thorough statistical measure to define a hit, using the Strictly Standardized Mean Difference (SSMD) method.

# Referee report: Reviewer #3 (Public review)

**DOI:** 10.7554/eLife.105314.3.sa3

Summary:

In "A‬‭ whole-animal‬‭ phenotypic‬‭ drug‬‭ screen‬‭ identifies‬‭ suppressors‬‭ of‬‭ atherogenic‬ lipoproteins", Kelpsch et al seek to identify new, chemically targetable pathways that regulate ApoB function and could ultimately serve as treatments for elevated lipid disorders and/or cardiovascular disease. Given the interconnected nature of lipid regulation in the whole organism with interdependent organs and secreted components (i.e. lipoproteins), they use the vertebrate model zebrafish to screen a large library of ~3000 compounds for their ability to lower the important ApoB-containing lipoproteins. They find 49 hits with 19 compounds passing a higher level of scrutiny, and focus on the role of enoxolone in modulating B-Ip levels at least partly through the HNF4alpha transcription factor and, putatively, through downstream cholesterol/lipid biosynthetic pathways.

Strengths:

The study uses a well-validated in vivo stain (LipoGlo) for measuring lipoproteins in the context of a developing whole organism with a quantitative read-out on a high-throughput platform, allowing for screening of thousands of compounds altering the complex metabolic/physiologic functions necessary for lipoprotein production.

The use of genetic mutant HNF4alpha to assign the mechanism of action to the prime candidate compound studied (enoxolone) is a powerful approach for this challenging aspect of chemical genetics studies.

# Author comment: Author response

**DOI:** 10.7554/eLife.105314.3.sa4

**Authors:** Daniel J Kelpsch, Liyun Zhang, James H Thierer, Adrian G Rivera Cruz, Kobe Koren, Urmi Kumar, Yuki Lin, Monica R Hensley, Mira Sohn, Jun O Liu, Thomas Lectka, Jeff S Mumm, Steven Arthur Farber

**Affiliations:** Johns Hopkins University Baltimore United States; Johns Hopkins University Baltimore United States; Johns Hopkins University Baltimore United States; Johns Hopkins University Baltimore United States; Johns Hopkins University Baltimore United States; Johns Hopkins University Baltimore United States; Johns Hopkins University Baltimore United States; Carnegie Institution for Science Baltimore, Maryland United States; Johns Hopkins University Baltimore United States; Johns Hopkins University School of Medicine Baltimore United States; Johns Hopkins University Baltimore United States; Johns Hopkins University Baltimore United States; Johns Hopkins University Baltimore, Maryland United States

The following is the authors’ response to the original reviews.

**eLife Assessment**

In this important study, the authors have performed a zebrafish drug screen to identify suppressors of atherogenic lipoproteins. They utilize a well-established LipoGlo assay to find molecules that modulate these lipoproteins, identifying 49 potential hits. They perform some validation experiments, including studies linking enoxolone to its likely inhibitory effect on a specific transcription factor, HNF4alpha. Overall, the results are convincing and robust, and will open up new areas of exploration for those investigators interested in in vivo lipid biology.

We appreciate the manuscript assessment and provide new data (see below) that significantly increases the “strength of the evidence”.

**Public Reviews:**

**Reviewer #1 (Public review):**

Strengths:

The authors performed a whole-organism chemical screen with over 3000 agents. Such screens are challenging, and the authors used strict criteria for determining hits. The conclusions of this study are well supported by the presented data.

Weaknesses:

There are areas within the study and writing that can be improved and extended, specifically within the gene expression studies.

We appreciate the Reviewer recognizing the strength of the data and the challenging nature of performing a whole animal small molecule screen. With regard the “Weaknesses”, as the Reviewer suggested we improved and clarified the text and “extended” the study by adding analysis and discussion of six additional hits (see Figure 3 and Sup. Fig.1) and modified the results section with the addition of over a page of text describing the additional phenotyping results:

“Secondary characterization of selected validated B-lp lowering hits.

To further evaluate the biological relevance and potential mechanisms of selected validated hits, we performed secondary analyses assessing total B-lp levels, larval morphology, and lipoprotein size distribution. Our lab previously defined the method to measure total B-lp levels from whole animals by using the homogenate of a single zebrafish larva [35]. We confirmed that treatment of animals with 4 µM pomiferin significantly reduced total B-lp levels measured from homogenates collected from whole animals after treatment (p = 8.6x10^-4^; Figure 3A). However, pomiferin treatment (4 µM) produced animals with reduced body length and lethality at higher doses, suggesting developmental toxicity may confound interpretation of its B-lp-lowering effect.

Treatment of animals with riboflavin tetrabutyrate (Figure 3B) and calcipotriene (Figure 3C) reduced total B-lp levels (p < 2x10^-16^) but did not affect larval morphology. A key feature of B-lps is their size, often a proxy for the total amount of lipid in the particle [35]. Particle size can impact the particle's lifetime (e.g. in metabolically healthy humans, small particles are cleared rapidly by the liver) [54–56]. Thus, we also assessed whether these compounds alter B-lp size distribution. Animals were treated for 48 h with vehicle, 5 µM lomitapide, or a drug of interest, and whole-animal homogenates were prepared and subjected to native polyacrylamide gel electrophoresis followed by chemiluminescent imaging. B-lps were classified into four classes based on gel migration: zero mobility (ZM), very low-density lipoproteins (VLDL), intermediate-density lipoproteins (IDL), and low-density lipoproteins (LDL) as previously described [35]. Lomitapide treatment effectively reduces VLDL particles and increases LDL particles [35] (Figure 3E), whereas riboflavin and calcipotriene did not affect lipoprotein classes. Thus, riboflavin tetrabutyrate and calcipotriene reduce total B-lp levels without overt developmental toxicity or changes in lipoprotein subclass distribution, suggesting they may act through mechanisms that decrease overall particle abundance rather than altering lipoprotein turnover or catabolism.

Although doxycycline treatment lowered B-lp levels in whole fixed animals in the primary screen and validation studies, we did not observe a reduction in total B-lps in whole-animal homogenates (Figure 3D). However, we detected a slight increase in VLDL levels (p < 2x10^-16^; Figure 3E), suggesting that doxycycline may alter lipoprotein composition or distribution rather than total particle abundance.

Alternatively, two structurally related compounds, thiethylperazine and prochlorperazine, at 4 µM significantly reduced (p < 1.4x10^-10^ and p < 1.2x10^-6^ respectively), B-lp levels measured from whole-animal homogenates (Figure 3F and 3H). Furthermore, both 8 µM thiethylperazine and 8 µM prochlorperazine increased relative LDL (p = 1.2x10^-3^ and p = 2.3x10^-3^, respectively) and decreased relative VLDL levels (p = 8.4x10^-4^ and p = 9.1x10^-4^, respectively; Figure 3G and 3I) suggesting a shift toward smaller lipoprotein particles and a potential alteration in lipid processing or clearance pathways. Together, these results highlight the diversity of mechanisms among validated hits, ranging from compounds that reduce total B-lp abundance without affecting B-lp class composition to those that shift B-lp class distribution, while also underscoring the importance of secondary assays to distinguish true B-lp modulators from those that likely produce a B-lp effect through generalized toxicity.

Enoxolone significantly reduces B-lps in the larval zebrafish.

Hit compounds were prioritized for follow-up studies based on reproducible dose-dependent responses, minimal toxicity as indicated by normal morphology over development, lack of direct NanoLuciferase inhibition, and the presence of literature suggesting potential links to lipid metabolism. One compound meeting these criteria was enoxolone, also known as 18β-Glycyrrhetinic acid, (Figure 2 Drug 20, Supplemental Table 1, Supplemental Figure 1T, Supplemental Figure 2L).”

The Discussion now has the following additional text:

“Further validation of these hits demonstrated a wide range of potential mechanisms of lipoprotein regulation. We identified hits that affected larval development, some hits that reduced total B-lp levels, and several structurally related compounds that directly reduced B-lp particle size (Figure 3).”

**Reviewer #2 (Public review):**

Strengths:

The study was methodical and robust, using a published and well-validated zebrafish LipoGlo model. The authors validated the hits from the screen independently and considered the possibility that some drugs may have been detected as false positive results due to effects on the enzymatic activity of NanoLuciferase; only one hit, verteporfin, was shown to be a false positive. Using LipoGlo-Electrophoresis, the authors are able to obtain extra insights into the ApoB-lipoprotein size/subclass distribution. They showed that while enoxolone treatment reduces total B-lps, there are no overt changes in B-lp size distribution compared to vehicle-treated animals, other than a slight increase in the zero mobility (ZM) fraction, which contains very large particles and/or tissue aggregates. In contrast, the positive control, lomitapide, does show a change in B-lp size distribution compared to vehicle-treated animals - an increase in frequency of LDLs (low-density lipoprotein), but a decrease in VLDLs (very low density lipoprotein). This study also assesses the LipoGlo-Electrophoresis profile of HNF4⍺ inhibitors. Work in the zebrafish larvae means that the effect on overall development and an entire vertebrate organism can also be assessed. Finally, the authors applied a thorough statistical measure to define a hit, using the Strictly Standardized Mean Difference (SSMD) method.

We appreciate that the Reviewer valued the rigour and robustness of our approach.

Weaknesses:

While the screen was thorough and well-validated, the authors missed a chance to provide a lot of extra significance to a wide range of readership. While the hits were thoroughly validated and displayed, the authors could have also presented the LipoGlo-Electrophoresis for all validated hits or at least a number of them. This would hugely increase the insights into these compounds. Also, the authors chose to validate and follow up a mechanism for Enoxolone, yet this hit was already known to modulate lipid metabolism through HNF4⍺, therefore, hugely limiting the impact of the paper. So what the authors have shown that is novel is only subtly added to this - consistent in vertebrate models, RNA sequencing of pathways, further validation of the HNF4⍺ pathway, and a profile of resulting B-lp size distribution. It seemed an easy way out to pick such a candidate, and they could have followed up by validating more thoroughly a completely novel drug. Also, the authors' prior paper showing the methodology also depicted complementary EM and LipoGlo-microscopy approaches. The microscopy especially, would have been an easy complementary add-on to the screen to really give extra insights into B-lp metabolism in a whole organism for all candidates. This felt like a missed opportunity.

Here we agree and added Fig. 3 describing the phenotyping of 6 additional compounds including some LipoGlo-Electrophoresis analyses as suggested by the Reviewer. The text of the Results section was modified as described for Reviewer 1 (see above).

**Reviewer #3 (Public review):**

Strengths:

The study uses a well-validated in vivo stain (LipoGlo) for measuring lipoproteins in the context of a developing whole organism with a quantitative read-out on a high-throughput platform, allowing for screening of thousands of compounds altering the complex metabolic/physiologic functions necessary for lipoprotein production.

The use of genetic mutant HNF4alpha to assign the mechanism of action to the prime candidate compound studied (enoxolone) is a powerful approach for this challenging aspect of chemical genetics studies.

We appreciate that the Reviewer understands how challenging it can be to assign a mechanism to any small molecule and thereby recognizes the power of the zebrafish model combined with our unique lipoprotein phenotyping tools.

Weaknesses:

As shown in Figure 5A, the HNF4alpha mutant homozygous -/- already lowers lipoproteins. Is it just that the mutant level is already at a minimum in this homozygous mutant (and thus enoxolone cannot induce even lower lipoprotein levels), or is it true that the enoxolone molecule is primarily acting through this TF (i.e. HNF4alpha homozygous mutant is truly epistatic to enoxolone function) as favored in the text.

While it is definitely interesting to study enoxolone effects during whole embryo development, the link to HNF4alpha had previously been described in the literature, as pointed out by the authors. The generalizability of the approach to identify truly novel pathways remains to be fully realized, but sharing this available screen data to date will invite further inquiry and be very valuable to the community.

Here too we agree that a link between enoxolone was proposed in the literature. However, we added quite a lot of additional insight regarding the transcriptional targets shared by HNF4alpha and enoxolone. The goal of identifying the mechanism(s) of action of other novel small molecule hits from the screen is important and that work is ongoing.

Figure 5 - The same allele of HNF4alpha loss of function/hypomorph (rdu14) is used in both 5A and 5B, but labeled differently in each subpanel. This is explained in the figure legend, but could be updated to use the same nomenclature in both panels to clarify the Figure presentation.

We thank the Reviewer for catching this and have modified the Figure (now Fig. 6) so the subpanels are labeled identically to avoid any confusion.

**Recommendations for the authors:**

**Reviewer #1 (Recommendations for the authors):**

(1) The authors describe statistical methods to improve the calling of hits. In the results section, they discuss the use of fold change and of strictly standardized mean difference as criteria to account for the variability that occurs within in vivo screens. The combination of both criteria resulted in a significant reduction in the total number of hits, from 487 (16%) to 49 (1.6%), which is a large decrease in total hits. The authors should comment on whether some of the 487 compounds were randomly tested individually to confirm that they were true negatives and compared to the true hits of 49. For example, did the authors independently test calcipotriene and diphenylboric acid to confirm that these are truly negative?

Initially, we did not directly retest any of the 487 compounds outside of the top 49 hits with the intention to compare their effect size to the top compound. While we do suspect that there is a chance that some of these compounds may still have a significant effect on lipoprotein levels, we prioritized compounds with the strongest overall biological effect size and largest statistically significant effect. We agree with the Reviewer that it would be interesting to continue to test more of these compounds and examine whether the lipoprotein reduction phenotype correlated with the primary screen effect, this would be a large experimental undertaking, though a randomized subset could be tested. Validation studies of Calcipotriene (Supplemental Figure 2E) showed a small, but significant, lipoprotein reduction at only the 0.25 µM dose across 3 independent experiments. Because the effect is small and not a dose-response, it is not a highly prioritized hit. We did not validate diphenlylboric acid in this study but will in the future.

(2) The transcriptomics profile studies are not well described. The authors do not provide a detailed list of differential genes for each of the conditions in their dataset. This should be included.

Supplemental Table 2 includes the fold change and p-values of all genes measured in differential expression analysis. We agree with the Reviewer and added new supplementary tables (Supplemental Table 5 and 7) that contain the differentially expressed genes at each treatment duration.

(3) The Gene Ontology analysis results are not accompanied by a list of genes that match the GO terms. The authors should include these results. This part was confusing as it was not clear if the cholesterol pathway affected was due to an abundance of genes that were up- or down-regulated.

We agree with Reviewer 1 and added Supplemental Table 6 that contains each gene ontology term with the associated DE genes that contribute to the significance of that term as well as explicit directionality information.

(4) Figure 6A shows heat maps of differential gene expression, but there is no key provided in the figure or legend. Are they color-coded for fold change or log2 fold change?

We agree that Figure 6A can be approved and added a key to the legend as suggested by the Reviewer.

(5) The overlap of genes that are changed in hnf4a mutants and with enoxolone is provided as percentages, but the actual genes are not listed. Do these genes represent cholesterol biosynthesis pathways? There are other bioinformatic tools that the authors could apply to their dataset for further analyses. For example, enrichr (https://maayanlab.cloud/Enrichr/) is one tool to query for GO Biological processes, cell/tissue type, and even overlap of genes with genetic models and other disease states. An extension of these bioinformatic studies will be useful to determine if other pathways are relevant.

We agree with the Reviewer and added a table of the genes that overlap between drug treatments and hnf4a mutants (Supplemental Table 7). We also appreciated the Reviewer’s suggestion to deploy Enrichr, which we have done and modified the Results section to now read:

“Of the 439 differentially expressed genes from 12, 16, and 24 hours post-treatment, 34 differentially expressed genes are shared between all three treatment durations and are associated with gene ontology terms related to carbohydrate metabolism and signaling pathways (Figure 7D). We expanded this analysis using the bioinformatic tool Enrichr [68–70], which largely recapitulated gene ontology results described above. However, Enrichr analysis revealed significant overlap between several late enoxolone-responsive gene sets and transcriptional signatures associated with prochlorperazine, another compound identified in our screen (Figure 2, Figure 3, Supplemental Figure 1AO, Supplemental Figure 2Z). Enrichment of prochlorperazine-associated signatures was observed at 12 (prochlorperazine MCF7 up, 4/58 genes [INSIG1;IRF7;ISG15;ATF3], adjusted p-value = 0.006), 16 (prochlorperazine MCF7 up, 6/58 genes [INSIG1;DDIT4;IRF7;PMAIP1;ISG15;ATF3], adjusted p-value = 0.00008; prochlorperazine PC3 up, 3/29 genes [INSIG1;DDIT4;ATF3], adjusted p-value = 0.006), and 24 hours post-treatment (prochlorperazine PC3 up, 6/29 genes [DUSP5;INSIG1;DDIT3;TRIB3;SQSTM1;ATF3], adjusted p-value = 0.0001; prochlorperazine MCF7 up, 7/58 genes [DDIT3;INSIG1;IRF7;PMAIP1;ISG15;SAT1;ATF3], adjusted p-value = 0.0005). These results suggest that enoxolone and prochlorperazine may perturb overlapping molecular pathways, an observation that warrants further investigation. Ultimately, these data demonstrate distinct early and late responses to enoxolone treatment, and the early response modulates key lipid metabolism pathways.”

(6) Figures 1E and 1F would benefit if the exact spots/points where enoxolone and other hits mentioned in the text were labelled.

Great idea, we modified Figure 1F as suggested.

(7) Figure 3 and Figure 4 graphs should state Fold change on the Y-axis title.

We are thankful the Reviewer noticed this typo and we have fixed both Figures.

**Reviewer #2 (Recommendations for the authors):**

(1) To boost the impact for more readers, the authors should include the LipoGloElectrophoresis and LipoGlo-Microscopy results from a few more of the validated hits, especially ones that are completely novel (unlike Enoxolone, which already had a known role in lipid metabolism). Results on enoxolone are useful as a validation of the assay, mostly with some minor additional insights.

We agreed with Reviewer 2 and added more validation testing of for a few hits (see response to Reviewer 1, Strengths and Reviewer 2, Weaknesses). We did not perform these experiments on each drug for technical reasons, mainly because these experiments are low-throughput (especially the Microscopy). Nonetheless, we performed many additional experiments to add phenotyping data for 6 new drugs that included multiple LipoGlo-Electrophoresis panels to an entirely new Figure.

(2) The authors should include raw data from the screen from all drugs tested in the supplementary and then for which SSMD was calculated for, providing in an excel sheet or similar the values and how these were calculated, i.e. the 487 unique drugs that lower B-lp levels with an SSMD cutoff of < -1.0.

This information was provided in the supplemental file as separate .csv files with the associated R script, which can be run locally and contains the SSMD functions. Considering the Reviewer comments, we ensured this information in provided in Supplemental Tables 1 and 2.

(3) Page 3, lines 23-24: What does the 2 to 4 fold chance mean? Perhaps rewrite: Genetic mutations in Lipoprotein(a) increase the chance of heart attack or stroke 2-4 fold greater than without the mutation.

We agree and the sentence now reads: Patients with genetic mutations in the Lipoprotein(a) encoding gene have a 2 to 4 fold increased risk of sudden heart attack or stroke.

(4) Figure 1, for C and D, label some of the most significant hits and definitely show where exonolone lies.

We agree see response to Reviewer 1 Pt6

(5) Page 6, lines 1-9: I'm a bit confused why this is here if you do not present the data.

We thought this was relevant information to share for researchers that running drug screens with positive controls and defining hit cutoffs. In light of the Reviewer’s comment, we removed the last sentence from this paragraph.

(6) Page 6, line 8: This needs better justification of why you are validating enoxolone rather than other hits; otherwise, it could seem like cherry picking. Especially as enoxolone is known to affect lipid metabolism. Otherwise present more details of a couple of validated candidates.

We agree. As the Reviewer requested, we validated more compounds (described above) and modified the text of the results to elaborate on our justification for selecting enoxolone for further study. The text of the results now reads: Hit compounds were prioritized for follow-up studies based on reproducible dose-dependent responses, minimal toxicity as indicated by normal morphology over development, lack of direct NanoLuciferase inhibition, and prior reports the presence of literature suggesting potential links to lipid metabolism. One compound meeting these criteria was enoxolone, also known as 18β-Glycyrrhetinic acid, (Figure 2 Drug 20, Supplemental Table 1, Supplemental Figure 1T, Supplemental Figure 2L).

(7) Supplementary Figures 1 and 2: The resolution is too low, and the reader cannot even see the charts or the text.

We agree and now have uploaded higher resolution images

(8) Page 7, lines 31-35: Needs a higher resolution and magnified image to merit this 'offhand' statement. Also cite reference [59] here.

We agree with the Reviewer and added magnified insets of the heart. As far as the suggestion of adding Ref 59, we do not see the connection to that paper (A point mutation decouples the lipid transfer activities of microsomal triglyceride transfer protein PLOS Genetics 16:e1008941)

(9) Figure 3E: In addition to the proportions graph would be useful to also have an absolute amount of lipoprotein in each class graph.

While there may be changes in total luminescence values from lane to lane in these gels, we have not fully validated the absolute quantitation of a full lane. We typically use plate-based whole-animal assays to determine total absolute lipoprotein levels and calculate the proportion of the whole lane for each lipoprotein class, as described in our prior publication detailing the assay. We do expect that there is some additional variation incorporated into the native PAGE assay due to sample freeze/thaw, dilution, and loading.

(1) Page 10 lines 27-28: "Continual statin use for more than 1 year reduced circulating Blps and all-cause mortality by ~30% in individuals with high B-lp levels." This sentence doesn't seem right, intimates continual statin use causes death - I don't think that's right.

We thank the Reviewer for catching this and have corrected the sentence. It now reads: “Continual statin use for more than 1 year in individuals with high B-lp levels reduced circulating Blps and lowered all-cause mortality by ~30% [12,13].”

(11) Page 12, line 25: "canlikely" is a typo, should be can likely.

We fixed that sentence and now reads: “Further, the drug screening paradigm we developed using the LipoGlo system is highly scalable and can be deployed to screen large novel drug libraries to identify many additional B-lp-lowering compounds.”

(12) Figure 1 legend: "An ordered plot of each SSMD score measured from 5 μM lomitapide treated animals from each 96-well plate (n = 1381) relative to respective vehicle treatment." This comes across as though it's 5uM Iomitapide/vehicle. But it's the SSMD score of each drug compared to Iomitapide and relative to the respective vehicle (I think) - make it clearer.

We agree and clarified the legend so it now reads: “…(D) An ordered plot of each SSMD score measured from positive control (5 µM lomitapide) treated animals from each 96-well plate (n = 1381) relative to respective vehicle treatment. The solid black line at y = 0 represents the divide in increased and decreased SSMD score, the solid blue line at y = -1.41 represents the curve's inflection point, and the dashed black line at y = -1 represents the SSMD (open circles) cutoff used to define a hit. “

(13) Figure 1E: What is the x axis?

Each data point on the x-axis represents each drug at every dose tested, we will clarify the test. The legend now reads: “(E) A plot of SSMD scores measured from each drug at each dose tested, each open circle represents the SSMD score of an individual drug at an individual dose.” In addition, “Compound (each dose tested)” was added to the x-axis of the figure panel.

(14) Figure 2: Would you not have space to put the drug names in the figures? Where, for example, is enoxolone?

We agree and have updated the figure accordingly.

(15) Figure 3A-C: label enoxolone on the x axis.

We agree and added this text to what is now Figure 4.

(16) Figure 3D: Looks like delayed development with enoxolone, if left to grow, would the embryos develop normally?

We did not examine if animals treated from 3-5 dpf develop normally beyond 5 dpf.

(17) Figure E. Are stars all compared to vehicle control? Perhaps useful to have lines to indicate what are the significantly different relationships.

Comparison in these experiments are always to the vehicle (negative control) and clarified the legend considering the Reviewer’s comment we modified the legend to now read,”… * <0.05 as compared to vehicle.”

(18) Figure 3E: As well as the proportion of total lipoprotein, it would also be beneficial to see absolute lipoprotein levels.

See above response to Reviewer 2 Pt9.

(19) Figure 4: Does the overall health or size of the animal correlate with the luminescence score?

While we do know that, in untreated animals, lipoprotein levels vary with age (and, thus, size), we have not examined this more granularly than in 24-hour time points after treatment. Further, we have not examined this in the context of a drug treatment.

(20) Figure 4D: Again the absolute in each fraction would be meaningful, also the 5078 looks brighter?

See above response to Reviewer 2 Pt9.

(21) Figure 4A, 5A: Would the traces (line plots) not be useful here to see the overall dynamics over time?

We considered presenting Figure 5A this way but decided to keep the plots as is because we wanted to show the individual points which make the line blots very difficult to read. Further, our analysis evaluates individual animals at each time point as it is not possible to follow the same animal over time.

**Reviewer #3 (Recommendations for the authors):**

Figure 4: Consider using standard scientific notation for the very small p values in some of the figure legends.

We agree and adjusted the p-value notation as suggested.
